# Predictors of Residual Pulmonary Vascular Obstruction after Acute Pulmonary Embolism Based on Patient Variables and Treatment Modality

**DOI:** 10.3390/jcm13144248

**Published:** 2024-07-20

**Authors:** Truong-An Andrew Ho, Jay Pescatore, Ka U. Lio, Parth Rali, Gerard Criner, Shameek Gayen

**Affiliations:** 1 Department of Thoracic Medicine and Surgery, Temple University Hospital, Philadelphia, PA 19140, USA; jay.pescatore@tuhs.temple.edu (J.P.); shameek.gayen@tuhs.temple.edu (S.G.); 2 Department of Medicine, Lewis Katz School of Medicine at Temple University, Philadelphia, PA 19140, USA

**Keywords:** pulmonary embolism, reperfusion, residual pulmonary vascular obstruction, chronic thromboembolic disease, catheter-directed therapy

## Abstract

**Background:** Residual Pulmonary Vascular Obstruction (RPVO) is an area of increasing focus in patients with acute pulmonary embolism (PE) due to its association with long-term morbidity and mortality. The predictive factors and the effect catheter-directed therapies (CDT) have on RPVO are still under investigation. **Methods:** This is a single-center retrospective review between April 2017 and July 2021. Patients with intermediate risk of PE were included. Patient variables associated with RPVO were analyzed and the degree of clot burden was quantified using the Qanadli score. **Results:** A total of 551 patients with acute PE were identified, 288 were intermediate risk and 53 had RPVO based on CT or V/Q scan three months post-PE. Baseline clot burden was higher in patients who received CDT compared to those who received anticoagulation alone (Qanadli score 45.88% vs. 31.94% *p* < 0.05). In univariate analysis, treatment with CDT showed a HR of 0.32 (95% CI 0.21–0.50, *p* < 0.001) when compared with anticoagulation alone. Patient variables including intermediate-high risk, sPESI ≥ 1, elevated biomarkers, RV dysfunction on imaging, malignancy, history of or concurrent DVT were also significantly associated with development of RPVO in univariate analysis. In multivariable analysis, only baseline Qanadli score (HR 13.88, 95% CI 1.42–135.39, *p* = 0.02) and concurrent DVT (HR 2.53, 95% CI 1.01–6.40, *p* = 0.04) were significantly associated with the development of RPVO. **Conclusions:** Catheter-directed therapy may be associated with a reduced risk of RPVO at 3 months; however, quantitative clot burden scores, such as the Qanadli score, may be stronger predictors of the risk of developing RPVO at 3 months. Further prospective studies are required

## 1. Introduction

Residual Pulmonary Vascular Obstruction (RPVO), defined as persistent vascular obstruction after a pulmonary embolism (PE), has been identified as a key pathophysiologic step in the development of chronic thromboembolic pulmonary hypertension (CTEPH) and chronic thromboembolic disease (CTED) [1]. It has also been associated with an increase in morbidity and mortality after acute pulmonary embolism [2,3], and recurrence of PE [4,5,6]. Currently, the factors that elevate a patients’ risk of RPVO remain under investigation. One study by Raj et al. used the data from the PADIS-PE trial and found that six months after a patients first unprovoked PE, age more than 65, pulmonary vascular obstruction index ≥ 25%, elevated factor 8 level, and chronic respiratory disease were predictors of RPVO [7]. Similarly, Sanchez et al. found that age, longer time between symptom onset and diagnosis, initial pulmonary vascular obstruction, and previous VTE (Venous Thromboembolism) were associated [8].

Given the elevated risk of bleeding with systemic thrombolytics, catheter-directed therapies (CDT) have become an increasingly studied treatment modality for intermediate-risk PE, providing an alternative to simple anticoagulation alone [9]. While CDT has been shown to have the potential to improve hemodynamics [10,11,12], more benefits in terms of patient centered outcomes remains to be seen. The ability for CDT to decrease rates of post PE syndromes such as RPVO is currently lacking in evidence. Defining these long-term benefits becomes increasingly important as the usage of CDT has risen sharply over the recent years and is expected to continue to rise [13].

We hypothesize that the treatment of intermediate-risk PE via CDT is associated with a reduced risk of RPVO after acute PE. Our primary objective was to determine significant and independent associations with the risk of developing RPVO after acute PE. 

## 2. Material and Methods

This is a single-center retrospective review of the electronic medical record between April 2017 and July 2021 for consecutive patients that were collected from the Pulmonary Embolism Response Team database. This study was performed in accordance with the ethical standards of the Helsinki Declaration of 1975 and Western Institutional Review Board (TEMP-9448).

Inclusion criteria included patients with acute pulmonary embolism who were intermediate, low or high, risk based on the European Society of Cardiology guidelines [14]. CT angiogram or ventilation perfusion imaging obtained at least 3 months after treatment were required to identify persistent clot burden. Patient variables identified were risk category per European Society of Cardiology, sPESI, Brain Natriuretic Peptide (BNP), troponin, right ventricular dysfunction on CT angiogram or transthoracic echocardiogram, active malignancy, concurrent deep vein thrombosis (DVT), prior VTE, and initial Qanadli score. The Qanadli score is a validated quantitative clot burden score. It is an additive point scale scoring system in which the maximum score is 40, such that the Qanadli embolism index = (embolism number × embolism degree)/40 × 100% [15]. An internal medicine physician was responsible for the calculation of the Qanadli score. Descriptive statistics with univariate and multivariable logistic regression were applied to determine associations with the risk of RPVO. Statistical significance was defined as a *p* valve less than 0.05 and a confidence interval that did not cross 1. ROC curves were created assessing the performance of the Qanadli score as a predictor of RPVO in patients that received CDT or anticoagulation alone. 

## 3. Results

We identified 551 patients with acute PE that were evaluated during our study period. Of those, 270 were intermediate-low, or high risk and were included in the analysis (Table 1). A total of 51% (*n* = 138) of patients were female, and mean age was 58.9 with a SD of 15.2. A total of 30% (n = 81) of patients had a history of VTE, and 20.7% (*n* = 56) had a history of malignancy. A total of 40.7% (*n* = 110) of patients were intermediate-low risk and 59.3% (*n* = 160) were intermediate-high risk. On admission, 63.8% (166/260) had an elevated BNP, and 51.7% (139/269) had an elevated troponin. In initial imaging studies, 67.7% (178/263) of patients who underwent CT scan showed RV dysfunction, while 73.8% (194/263) of patients who underwent echocardiogram showed RV dysfunction. A total of 57.4% (143/249) of patients who had lower extremity dopplers performed were positive for DVT. 

In regard to treatment, 53% (*n* = 143) were treated with anticoagulation (AC) alone, 44.8% (*n* = 121) were treated with AC in conjunction with additional therapy, and 2.2% (*n* = 6) received no AC. Of those patients that received advanced therapies, 74.2% (*n* = 89) received CDT, 9.9% (*n* = 12) received systemic thrombolysis, 16.5% (*n* = 20) received mechanical thrombectomy, and 2.4% (*n* = 3) received surgical thrombectomy. There were 53 patients who had RPVO based on either CT or V/Q scan three months after acute PE (Table 1).

For the degree of clot burden, the Qanadli score at baseline for all intermediate-risk PE patients had a mean of 38.87% with a SD of 20.5%. The baseline Qanadli score for patients with RPVO had a mean of 51.18% with a SD of 11.9%, while patients without RPVO had a mean of 34.97% with a SD 21.2%. In patients who underwent CDT, the mean baseline Qanadli score was 45.88% with a of SD 19.6% as compared to the group that received AC alone, which had a Qanadli score of 31.94% with a of SD 16.9%. *T*-test between mean CDT Qanadli score and anticoagulation group alone was <0.05.

Univariate logistic regression was performed to assess factors associated with RPVO at 3 months post-PE; variables with significant association were then utilized in multivariable logistic regression to determine independent and significant associations with risk of RPVO. Treatment with CDT and AC showed a reduced risk of RPVO development (HR 0.32, 95% CI 0.21–0.50, *p* < 0.001) when compared with AC alone. Patient variables including intermediate-high risk, sPESI greater than or equal to 1, elevated biomarkers (BNP or troponin), RV dysfunction on imaging (CTA or echocardiogram), and concurrent DVT were also significantly associated with development of RPVO in univariate analysis. In multivariable analysis, only the baseline Qanadli score (HR 13.88, 95% CI 1.42–135.39, *p* = 0.02) and concurrent DVT (HR 2.53, 95% CI 1.01–6.40, *p* = 0.04) were independently and significantly associated with an increased risk of the development of RPVO (Table 2).

**Figure 1:**
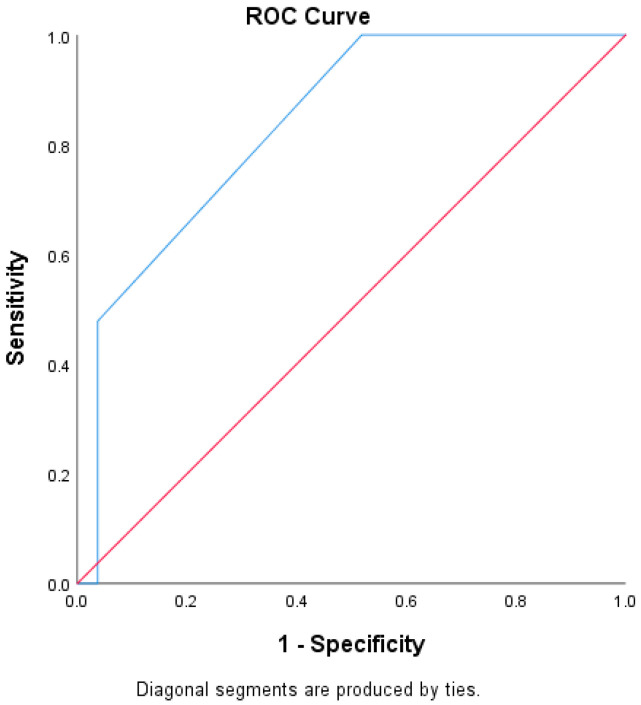
ROC curve assessing the Qanadli score as a predictor of RPVO (Residual Pulmonary Vascular Obstruction) in patients who received CDT (catheter directed therapy). AUC 0.84, *p* < 0.001.

**Figure 2:**
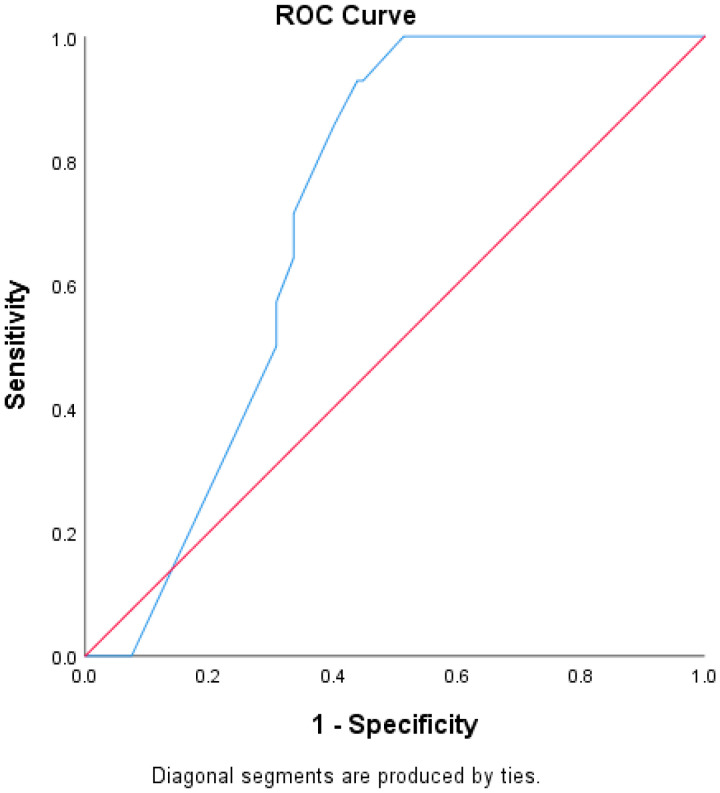
ROC curve assessing the Qanadli score as a predictor of RPVO(Residual Pulmonary Vascular Obstruction) in patients who received AC (anticoagulation) alone. AUC 0.72, *p* < 0.001.

The area under the ROC curve (AUC) was used to compare the ability of the Qanadli score to predict RPVO in patients who received CDT and those who received AC. The AUC for the CDT group was 0.84, *p* < 0.001 and the AUC for the patients who received AC alone was 0.72, *p* < 0.001 (Figure 1 and Figure 2).

## 4. Discussion

Post-pulmonary embolism syndromes, including CTEPH, CTED, and RPVO, are an area of increased investigation due to their long-term morbidity and mortality, necessitating studies into predictive factors [2,3]. Many patient characteristics have been associated with clot resolution including initial RV/LV diameter, PE location and size, degree of obstruction, early initiation of treatment, and provoked vs. unprovoked status [16,17]. Our findings noted significantly increased RPVO risk in patients with intermediate-high-risk PE, sPESI greater than or equal to 1, elevated biomarkers, RV dysfunction on imaging, and concurrent DVT. In multivariable analysis, however, only the degree of baseline clot burden, as measured by the Qanadli score and concurrent DVT, remained significantly associated with the development of RPVO. ROC were performed to assess the effectiveness of the Qanadli score and it was shown to perform well as a predictor of RPVO in both the CDT and AC groups, though slightly better in the CDT group.

The degree of initial clot burden has been consistently shown to be a risk factor for lack of clot resolution [7,8,16,17]. This suggests that quantifying the degree of clot burden, whether by Qanadli score, or other objective measurement allows for risk stratification of patients who may go on to develop post-PE syndromes. The Qanadli score is labor intensive, but with increased use of artificial intelligence in the workup of pulmonary embolism, rapid estimation of clot burden may be possible in the future. A small study by Sun et al. in 2020 found that computer-aided interpretation of vascular obstruction, as measured by the Qanadli score, reduced the time for interpretation and reliability of radiologists’ findings [18]. PE identification pathways have already seen the integration of artificial intelligence in other ways, outside of clot burden measurements, and has shown synergistic effects when implemented alongside standard radiology practices [19,20]. Larger studies will be needed to see if initial clot burden scores can be integrated into risk stratification.

The increased risk of RPVO in intermediate-risk PE patients with concurrent DVT was also significant in our study. DVT associated with PE has been shown to be associated with increased PE-specific and all-cause mortality [21,22,23] and has been considered in the risk stratification of PE [24]. In RPVO, however, prior studies have not shown a statistically significant relationship between DVT and residual clot burden [5,6,8]. It is unclear the explanation for this; however, these prior studies assessed all-comers for PE and may have included a subset of patients in which clot resolution occurred without issue, while our intermediate-risk patients may have a higher proportion of patients that have problems resolving clot. We propose that having a DVT at time of diagnosis represents an elevated degree of clot burden, similar to a higher Qanadli score. Further studies will need to reassess the effects of DVT in specific PE populations in respect to RPVO, given the conflicting findings.

The role of CDT in intermediate-risk PE remains under investigation, and current guidelines suggest their use only in patients who have a contraindication or who have failed systemic thrombolysis [14,25]. Small prospective studies have focused on CDT’s ability to improve RV dysfunction, as measured by the RV/LV ratio, without increased bleeding [10,11,12]; however, data showing long term benefit is lacking. A case series in 2022 by Gayen et al. examined three patients who underwent mechanical thrombectomy, and measured pre and post procedure perfusion scores, finding sustained improvements in perfusion immediately post procedure and at 3 months [26]. This suggests that other advanced therapies can improve long-term perfusion, but further studies focused on catheter-directed therapy are needed especially as their usage increases [13]. 

In our study, while CDT was associated with a reduced risk of RPVO on initial univariate analysis, it was not statistically significant in the multivariable analysis; however, there was a trend towards reduced risk (HR 0.35, 95% CI 0.11–1.12, *p* = 0.08). One potential explanation for this is that patients selected to undergo CDT had higher initial clot burden as compared to those who did not (Qanadli score 45.88% vs. 31.94%), and it is that initial clot burden that has the highest association with RPVO. Another possibility is that our sample size was too small; however, the potential benefit of CDT in reducing RPVO demonstrated in our analysis is promising. The ROC assessment demonstrated the increased effectiveness of the Qanadli score to predict RPVO in patients who received CDT as compared to AC; however, further studies are warranted (AUC 0.84, *p* < 0.001 vs. 0.72, *p* < 0.001) (Figure 1 and Figure 2). To minimize confounders, further studies will require stratifying patients to different treatment arms based on their baseline clot score to truly examine the effects of catheter-directed treatments on long-term, patient-centered outcomes.

Limitations to this study include its retrospective nature, and while we are not able to show causation, our findings are consistent with prior studies evaluating predictive factors of clot nonresolution. Follow-up imaging was not performed at standard time periods; however, all imaging was collected after at least three months of therapy. It may also be significant that the majority of the patients in our study who received advanced therapies received catheter directed thrombolysis (74.2%) and relatively few received mechanical thrombectomy. The composition of clots seen in pulmonary embolism have been shown to evolve over time and have both different macro and micro appearances, with acute clots having higher fibrin content while more chronic clots consist of higher portions of collagen and elastin [1,27]. The chronicity of the clot has also been associated with resistance to fibrinolytics in other populations such as arterial thrombi and deep vein thrombosis [27,28]. This suggests that there may be a subset of patients with more chronic organized clot that may have improved response to mechanical removal given their decreased responsiveness to fibrinolytics; however, that remains to be proven, and improvement in CT imaging ability to distinguish chronicity would be required to stratify patients. The Qanadli score was also calculated by one of the authors, an internal medicine physician. While we believe this limited interoperator variability, future studies, in which a radiologist or artificial intelligence system are utilized, are warranted. Also challenging is how we define post-PE syndromes, such as RPVO, CTED, and CTEPH, with language that continues to evolve. For that reason, our study focuses solely on clot nonresolution, without defining downstream disease processes; however, future studies will be needed.

In conclusion, the degree of baseline clot burden, as measured by the Qanadli score, was the strongest predictor of RPVO after 3 months of therapy. Quantifying clot burden on admission and providing an objective measurement may allow the stratification of patients more likely to develop CTEPH or CTED. CDT may be associated with reduced risk of developing RPVO after acute intermediate-risk PE, but further prospective studies are needed.

## Figures and Tables

**Table 1:** Baseline demographics of intermediate risk PE patients, stratified by RPVO and treatment modality.

	Intermediate Risk PE *n* = 270	RPVO (+) *n* = 53	RPVO (−) *n* = 217	AC Alone *n* = 143	CDT *n* = 89
Demographic data					
Age—years	58.9 ± 15.2	59.2 ± 15.4	58.8 ± 15.2	59.8 ± 15.2	57.4 ± 13
Sex—no. (%)					
Female	138 (51.1)	33 (62.3)	105 (48.4)	64 (44.8)	60 (67.4)
Male	132 (48.9)	20 (37.7)	112 (51.6)	79 (55.2)	29 (32.6)
Race—no. (%)					
Caucasian	147 (54.4)	10 (18.9)	45 (20.7)	23 (16.1)	22 (24.7)
African American	55 (20.4)	33 (62.3)	114 (52.5)	76 (53.1)	48 (53.9)
Asian/Pacific Islander	64 (23.7)	6 (11.3)	58 (26.7)	42 (29.4)	17 (19.1)
Other/unknown	4 (1.5)	4 (7.5)	4 (1.8)	2 (1.4)	2 (2.2)
BMI	34.1 ± 10.5	30.9 ± 7.5	34.8 ± 10.9	32.2 ± 10.78	38.14 ± 9.6
Medical History					
History of DVT—no. (%)	58 (21.5)	8 (15.1)	50 (23)	32 (22.4)	20 (22.5)
History of PE—no. (%)	53 (19.6)	6 (11.3)	47 (21.7)	25 (17.5)	25 (28.1)
History of malignancy—no. (%)	56 (20.7)	10 (18.9)	46 (21.1)	34 (23.8)	11 (12.4)
History of COPD—no. (%)	27 (10)	4 (7.5)	23 (10.6)	19 (13.3)	6 (6.7)
History of recent surgery—no. (%)	29 (10.7)	4 (7.5)	25 (11.5)	19 (13.3)	0 (0)
Clinical Status					
sPESI—mean ± SD	1.58 ± 1.1	1.37 ± 1.07	1.62 ± 1.07	1.71 ± 1.06	1.1 ± 0.85
Intermediate-low risk—no. (%)	110 (40.7)	16 (30.2)	94 (43.3)	90 (63)	9 (10.1)
Intermediate-high risk—no. (%)	160 (59.3)	37 (69.8)	123 (56.7)	53 (37)	80 (89.9)
Oxygen treatment—no. (%)	136 (50.3)	29 (54.7)	107 (49.3)	70 (49)	44 (49.4)
Elevated BNP—no. (%)	166 (61.48)	37 (69.8)	129 (59.4)	78 (54.4)	65 (73)
Elevated troponin—no. (%)	139 (51.5)	31 (58.4)	108 (49.7)	55 (38.5)	60 (67)
RV dysfunction on CT—no. (%)	178 (65.9)	47 (88.7)	131 (60.4)	76 (53.1)	76 (85)
RV dysfunction on ECHO—no. (%)	194 (71.9)	41 (77.4)	153 (70.5)	95 (66.4)	74 (83.1)
Concurrent DVT—no. (%)	143 (57.4)	35 (66)	108 (49.8)	53 (37)	66 (74)
Therapy					
Anticoagulation alone—no. (%)	143 (53)	22 (41.5)	121 (55.5)	143 (100)	0 (0)
Catheter-Directed Thrombolysis—no. (%)	89 (33)	23 (43.3)	66 (30.3)	0 (0)	89 (100)
Mechanical thrombectomy—no. (%)	20 (7.4)	6 (11.3)	14 (6.4)	0 (0)	0 (0)
Systemic thrombolysis—no. (%)	12 (4.4)	2 (3.7)	10 (4.6)	0 (0)	0 (0)
Surgical thrombectomy—no. (%)	3 (1.1)	0 (0)	3 (1.4)	0 (0)	0 (0)
No therapy—no. (%)	6 (2.2)	0 (0)	6 (2.8)	0 (0)	0 (0)
Measurements of clot					
Baseline Qanadli score %	38.87 ± 20.5	51.18 ± 11.9	34.97 ± 21.2	31.94 ± 16.9	45.88 ± 19.5
+RPVO—no. (%)	53 (19.6)	53 (100)	0 (0)	22 (15.4)	23 (25.8)
−RPVO—no. (%)	217 (80.4)	0 (0)	217 (100)	121 (84.6)	66 (74.2)

PE, pulmonary embolism; RPVO, residual pulmonary vascular obstruction; AC, anticoagulation; CDT, catheter directed therapies; BMI, body mass index; DVT, deep vein thrombosis; COPD, chronic obstructive pulmonary disease; sPESI, simplified pulmonary embolism severity index; BNP, brain natriuretic peptide; RV, right ventricular; CT, computed tomography; ECHO, echocardiogram.

**Table 2:** Univariate and multivariate analysis of factors predictive of RPVO.

Patient Variables/Interventions	Univariate Analysis	Multivariate Analysis
AC + CDT	**HR 0.32, 95% CI 0.21–0.50, *p* < 0.001**	HR 0.35, 95% CI 0.11–1.12, *p* = 0.08
AC + tpa, mechanical thrombectomy	HR 0.40, 95% CI 0.13–1.28, *p* = 0.12	HR 1.99, 95% CI 0.58–6.77, *p* = 0.27
Intermediate-high risk	**HR 0.32, 95% CI 0.23–0.46, *p* < 0.001**	HR 0.98, 95% CI 0.28–3.42, *p* = 0.97
sPESI	**HR 0.55, 95% CI 0.45–0.66, *p* < 0.001**	HR 0.74, 95% CI 0.47–1.16, *p* = 0.19
Elevated biomarkers (BNP or troponin)	**HR 0.26, 95% CI 0.19–0.36, *p* < 0.001**	HR 0.96, 95% CI 0.29–3.19, *p* = 0.95
RV dysfunction on imaging (TTE or CTA)	**HR 0.** **28** **, 95% CI 0.** **20–** **0.** **39** **, *p* < 0.** **001**	HR 0.55, 95% CI 0.15–2.11, *p* = 0.39
Concurrent DVT	**HR 0.** **29** **, 95% CI 0.** **20–** **0.** **43** **, *p* < 0.05**	**HR 2.53** **, 95% CI 1.01–6.40** **, *p* = 0.** **04**
Baseline Qanadli score	**HR 6.58, 95% CI 1.35–32.17** **, *p* < 0.05**	**HR 16.12, 95% CI 2.47–20.84, *p* < 0.001**

Bolded font is statistically significant hazard ratio (HR). AC, anticoagulation; CDT, catheter directed therapies; tpa, tissue plasminogen activator; sPESI, simplified pulmonary embolism severity index; BNP, brain natriuretic peptide; TTE, transthoracic echocardiogram; CTA, computed tomography angiography; DVT, deep vein thrombosis.

## Data Availability

The data that support the findings of this study are available from the corresponding author upon reasonable request.

## Abbreviations

RPVO, Residual Pulmonary Vascular Obstruction); PE, pulmonary embolism; CTEPH, chronic thromboembolic pulmonary hypertension; CTED, chronic thromboembolic disease; VTE, Venous Thromboembolism; CDT, catheter-directed therapies; DVT, deep vein thrombosis; AC, anticoagulation; BMI, body mass index; COPD, chronic obstructive pulmonary disease; sPESI, simplified pulmonary embolism severity index; BNP, brain natriuretic peptide; RV, right ventricular; CT, computed tomography; ECHO, echocardiogram; TTE, transthoracic echocardiogram; CTA, computed tomography angiography; DVT, deep vein thrombosis.

## References

[1] Simonneau G., Torbicki A., Dorfmüller P., Kim N. (2017). The pathophysiology of chronic thromboembolic pulmonary hypertension. Eur. Respir. Rev..

[2] Meneveau N., Ider O., Seronde M.-F., Chopard R., Davani S., Bernard Y., Schiele F. (2013). Long-term prognostic value of residual pulmonary vascular obstruction at discharge in patients with intermediate- to high-risk pulmonary embolism. Eur. Heart J..

[3] Bonnefoy P.B., Margelidon-Cozzolino V., Catella-Chatron J., Ayoub E., Guichard J.B., Murgier M., Bertoletti L. (2019). What’s next after the clot? Residual pulmonary vascular obstruction after pulmonary embolism: From imaging finding to clinical consequences. Thromb. Res..

[4] Tromeur C., Sanchez O., Presles E., Pernod G., Bertoletti L., Jego P., Duhamel E., Provost K., Parent F., Robin P. (2018). Risk factors for recurrent venous thromboembolism after unprovoked pulmonary embolism: The PADIS-PE randomised trial. Eur. Respir. J..

[5] Planquette B., Ferré A., Peron J., Vial-Dupuy A., Pastre J., Mourin G., Emmerich J., Collignon M.-A., Meyer G., Sanchez O. (2016). Residual pulmonary vascular obstruction and recurrence after acute pulmonary embolism. A single center cohort study. Thromb. Res..

[6] Pesavento R., Filippi L., Palla A., Visonà A., Bova C., Marzolo M., Porro F., Villalta S., Ciammaichella M., Bucherini E. (2017). Impact of residual pulmonary obstruction on the long-term outcome of patients with pulmonary embolism. Eur. Respir. J..

[7] Raj L., Robin P., Le Mao R., Presles E., Tromeur C., Sanchez O., Pernod G., Bertoletti L., Jego P., Leven F. (2019). Predictors for Residual Pulmonary Vascular Obstruction after Unprovoked Pulmonary Embolism: Implications for Clinical Practice—The PADIS-PE Trial. Thromb. Haemost..

[8] Sanchez O., Helley D., Couchon S., Roux A., Delaval A., Trinquart L., Collignon M.A., Fischer A.M., Meyer G. (2010). Perfusion defects after pulmonary embolism: Risk factors and clinical significance. J. Thromb. Haemost..

[9] Pietrasik A., Gasecka A., Kotulecki A., Karolak P., Araszkiewicz A., Darocha S., Grabowski M., Kurzyna M. (2023). Catheter-directed therapy to treat intermediateand high-risk pulmonary embolism: Personal experience and review of the literature. Cardiol. J..

[10] Kucher N., Boekstegers P., Müller O.J., Kupatt C., Beyer-Westendorf J., Heitzer T., Tebbe U., Horstkotte J., Müller R., Blessing E. (2014). Randomized, Controlled Trial of Ultrasound-Assisted Catheter-Directed Thrombolysis for Acute Intermediate-Risk Pulmonary Embolism. Circulation.

[11] Piazza G., Hohlfelder B., Jaff M.R., Ouriel K., Engelhardt T.C., Sterling K.M., Jones N.J., Gurley J.C., Bhatheja R., Kennedy R.J. (2015). A Prospective, Single-Arm, Multicenter Trial of Ultrasound-Facilitated, Catheter-Directed, Low-Dose Fibrinolysis for Acute Massive and Submassive Pulmonary Embolism. JACC Cardiovasc. Interv..

[12] Bashir R., Foster M., Iskander A., Darki A., Jaber W., Rali P.M., Lakhter V., Gandhi R., Klein A., Bhatheja R. (2022). Pharmacomechanical Catheter-Directed Thrombolysis with the Bashir Endovascular Catheter for Acute Pulmonary Embolism. JACC Cardiovasc. Interv..

[13] Sedhom R., Megaly M., Elbadawi A., Elgendy I.Y., Witzke C.F., Kalra S., George J.C., Omer M., Banerjee S., Jaber W.A. (2022). Contemporary National Trends and Outcomes of Pulmonary Embolism in the United States. Am. J. Cardiol..

[14] Konstantinides S.V., Meyer G., Becattini C., Bueno H., Geersing G.-J., Harjola V.-P., Huisman M.V., Humbert M., Jennings C.S., Jiménez D. (2019). 2019 ESC Guidelines for the diagnosis and management of acute pulmonary embolism developed in collaboration with the European Respiratory Society (ERS): The Task Force for the diagnosis and management of acute pulmonary embolism of the European Society of Cardiology (ESC). Eur. Respir. J..

[15] Fink M.A., Mayer V.L., Schneider T., Seibold C., Stiefelhagen R., Kleesiek J., Weber T.F., Kauczor H.-U. (2022). CT Angiography Clot Burden Score from Data Mining of Structured Reports for Pulmonary Embolism. Radiology.

[16] Choi K.-J., Cha S.-I., Shin K.-M., Lim J.-K., Yoo S.-S., Lee J., Lee S.-Y., Kim C.-H., Park J.-Y., Lee W.-K. (2016). Factors determining clot resolution in patients with acute pulmonary embolism. Blood Coagul. Fibrinolysis.

[17] Aranda C., Gonzalez P., Gagliardi L., Peralta L., Jimenez A. (2021). Prognostic factors of clot resolution on follow-up computed tomography angiography and recurrence after a first acute pulmonary embolism. Clin. Respir. J.

[18] Sun Z.-T., Hao F.-E., Guo Y.-M., Liu A.-S., Zhao L. (2020). Assessment of Acute Pulmonary Embolism by Computer-Aided Technique: A Reliability Study. Med. Sci. Monit..

[19] Batra K., Xi Y., Al-Hreish K.M., Kay F.U., Browning T., Baker C., Peshock R.M. (2022). Detection of Incidental Pulmonary Embolism on Conventional Contrast-Enhanced Chest CT: Comparison of an Artificial Intelligence Algorithm and Clinical Reports. Am. J. Roentgenol..

[20] Cheikh A.B., Gorincour G., Nivet H., May J., Seux M., Calame P., Thomson V., Delabrousse E., Crombé A. (2022). How artificial intelligence improves radiological interpretation in suspected pulmonary embolism. Eur. Radiol..

[21] Jiménez D., Aujesky D., Díaz G., Monreal M., Otero R., Martí D., Marín E., Aracil E., Sueiro A., Yusen R.D. (2010). Prognostic Significance of Deep Vein Thrombosis in Patients Presenting with Acute Symptomatic Pulmonary Embolism. Am. J. Respir. Crit. Care Med..

[22] Becattini C., Cohen A.T., Agnelli G., Howard L., Castejón B., Trujillo-Santos J., Monreal M., Perrier A., Yusen R.D., Jiménez D. (2016). Risk Stratification of Patients with Acute Symptomatic Pulmonary Embolism Based on Presence or Absence of Lower Extremity DVT. Chest.

[23] Nishiwaki S., Morita Y., Yamashita Y., Morimoto T., Amano H., Takase T., Hiramori S., Kim K., Oi M., Akao M. (2021). Impact of no, distal, and proximal deep vein thrombosis on clinical outcomes in patients with acute pulmonary embolism: From the COMMAND VTE registry. J. Cardiol..

[24] Quezada C.A., Bikdeli B., Barrios D., Morillo R., Nieto R., Chiluiza D., Barbero E., Guerassimova I., García A., Yusen R.D. (2018). Assessment of coexisting deep vein thrombosis for risk stratification of acute pulmonary embolism. Thromb. Res..

[25] Stevens S.M., Woller S.C., Kreuziger L.B., Bounameaux H., Doerschug K., Geersing G.-J., Huisman M.V., Kearon C., King C.S., Knighton A.J. (2021). Antithrombotic Therapy for VTE Disease. Chest.

[26] Gayen S., Upadhyay V., Kumaran M., Bashir R., Lakhter V., Panaro J., Criner G., Dadparvar S., Rali P. (2022). Changes in Lung Perfusion in Patients Treated with Percutaneous Mechanical Thrombectomy for Intermediate-Risk Pulmonary Embolism. Am. J. Med..

[27] Chernysh I.N., Nagaswami C., Kosolapova S., Peshkova A.D., Cuker A., Cines D.B., Cambor C.L., Litvinov R.I., Weisel J.W. (2020). The distinctive structure and composition of arterial and venous thrombi and pulmonary emboli. Sci. Rep..

[28] Czaplicki C., Albadawi H., Partovi S., Gandhi R.T., Quencer K., Deipolyi A.R., Oklu R. (2017). Can thrombus age guide thrombolytic therapy?. Cardiovasc. Diagn. Ther..

